# Mechanosynthesis of Photochromic Oligophenyleneimines: Optical, Electrochemical and Theoretical Studies

**DOI:** 10.3390/molecules24050849

**Published:** 2019-02-28

**Authors:** Miguel Angel Amado-Briseño, Luis Ángel Zárate-Hernández, Karina Alemán-Ayala, Oscar Coreño Alonso, Julián Cruz-Borbolla, José Manuel Vásquez-Pérez, Víctor Esteban Reyes-Cruz, María Aurora Veloz-Rodríguez, Esteban Rueda-Soriano, Thangarasu Pandiyan, Rosa Angeles Vázquez-García

**Affiliations:** 1 Área Académica de Ciencias de la Tierra y Materiales, Área Académica de Computación y Electrónica, Área Académica de Química, Universidad Autónoma del Estado de Hidalgo, Ciudad Universitaria, Pachuca CP 42184, Mexico; am111261@uaeh.edu.mx (M.A.A.-B.); luiszarate93@hotmail.com (L.Á.Z.-H.); karina_aleman@uaeh.edu.mx (K.A.-A.); jcruz@uaeh.edu.mx (J.C.-B.); josemanuel_vasquez@uaeh.edu.mx (J.M.V.-P.); reyescruz16@yahoo.com (V.E.R.-C.); mveloz@uaeh.edu.mx (M.A.V.-R.); estebanrs@uaeh.edu.mx (E.R.-S.); 2 Dpto. Ing. Civil, Universidad de Guanajuato, Juárez 77, Guanajuato CP 36000, Mexico; ocoreno@yahoo.com; 3 Facultad de Química, UNAM, Cd. Universitaria, Circuito exterior, Coyoacán, México D.F. CP 04510, Mexico; pandiyan@unam.mx

**Keywords:** mechanochemistry, oligophenyleneimines, photochromics, theoretical calculations

## Abstract

In this work, two oligophenyleneimines type pentamers with terminal aldehydes, designated as DAFCHO (4,4′-((((((2,5-bis(octyloxy)-1,4-phenylene)bis(methanylylidene))bis(azanyl ylidene))bis(9H-fluorene-7,2-diyl))bis(azanylylidene))bis(methanylylidene))bis(2,5-bis(octyloxy) benzaldehyde)) and FDACHO (4,4′-((((((2,5-bis(octyloxy)-1,4-phenylene)bis(methanylylidene))bis (azanylylidene))bis(4,1-phenylene))bis(azanylylidene))bis(methanylylidene))bis(2,5-bis(octyloxy) benzaldehyde)) were synthesized by mechanochemistry method using 2,5-bis(octyloxy) terephtal aldehyde and 2,7-diaminofluorene or 1,4-phenylenediamine. All compounds were spectroscopically characterized using ^1^H and ^13^C-NMR, FT-IR and mass spectrometry MALDITOF. The optical properties of the compounds were analyzed by UV-vis spectroscopy using different solvents. We observed that DAFCHO and FDACHO exhibit interesting photochromic properties when they are dissolved in chloroform and exposed to sunlight for 3, 5 and 10 min. The value of the energy band gap was calculated from the absorption spectra without irradiation E_gap(optical)_. It was 2.50 eV for DAFCHO in chloroform solution, and it decreased to 2.34 eV when it is in films. For FDACHO, it was 2.41 eV in solution and 2.27 eV in film. HOMO (Highest Occupied Molecular Orbital), LUMO (Lowest Unoccupied Molecular Orbital) and E_gap(electrochemical)_ values were obtained by electrochemical studies. The results indicate that the compounds can be considered as organic semiconductors since their values are 2.35 eV for DAFCHO and 2.06 eV for FDACHO. The structural and electronic properties of the compounds were corroborated with a DFT (Density Functional Theory) study.

## 1. Introduction

The study of organic semiconductor compounds with photochromic properties is of great significance [1] in the manufacture of devices such as antireflective systems for screens [2], optical memory systems [3], optical switches [4,5,6], OFETs (Organic Field Effect Transistor) [7,8], smart windows [9] and a wide variety of sensors [10,11,12]. It has been shown that the photochromic response of organic materials depends on the functional groups type and on the molecule structure [1,13,14,15,16]. In previous studies, we reported on the reversible photochromic effect of imine-type materials, and we also reported on the type of terminal functional groups, as well as the synthesis method influencing these properties. [15,16,17,18]. A change in color is observed when imine type compounds were irradiated with sunlight; interestingly, the original color is recovered if the compound is placed in dark conditions. In this case, the π−π* transition undergoes a substantial change with respect to a configurational change in the molecules (*trans*-*cis photoisomerization*) [18,19]. This effect, observed on dyes with fluorene, thiophene, pyrrole and azole heterocycles, has been reported [20,21]. However, the synthesis of these compounds through traditional method [13] involves solvents, long reaction time and catalysts, generating high production costs. A technique, such as mechanochemistry, that is free from solvents and catalysts and environmentally friendly would make these materials attractive for industrial production. This mechanochemistry technique has been successfully employed to obtain organic compounds that exhibit attractive optoelectronic properties [22] and has been proved to be efficient for obtaining imine oligomers [15].

Organic imine-type materials are of interest in biotechnology [23], medicine [24] and considered as semiconductors for optoelectronic devices because of their low band gap energy of around 2.3 eV [25,26]. It is known that the incorporation of aliphatic chains in organic molecules increases the solubility [27] and structural planarity [28], allowing the material to form homogeneous films when manufacturing optoelectronic devices.

Two conjugated oligophenyleneimines pentamers were synthesized by means of the mechanochemical method and characterized by spectroscopic methods. The optical, electronic and electrochemical properties of the compounds were analyzed. Density Functional Theory (DFT) was used to interpret the molecular structural change relating to optoelectronic and chemical properties.

## 2. Results and Discussion

### 2.1. Synthesis and Characterization

**Figure 1:**
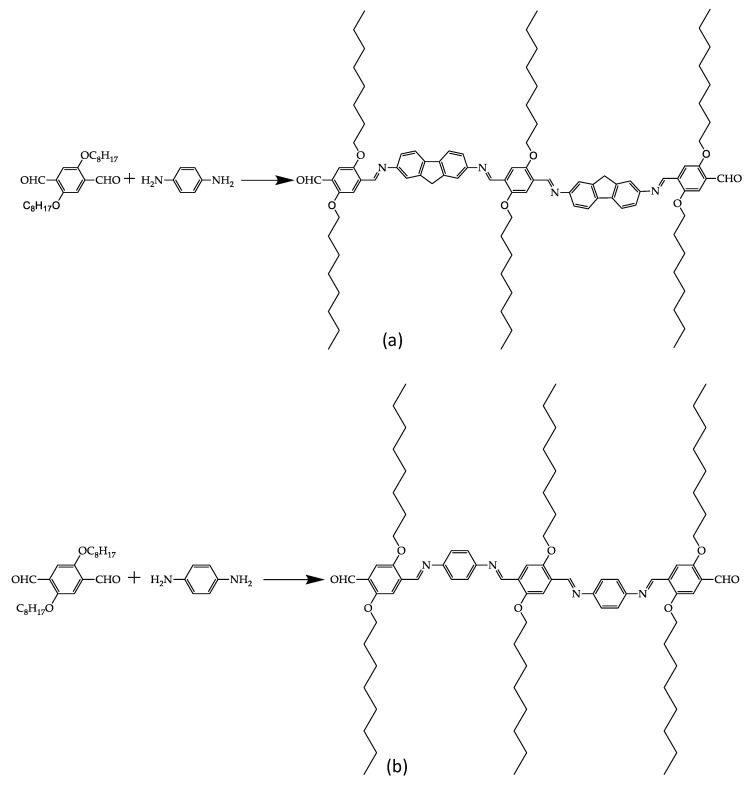
Synthesis of conjugated oligophenyleneimines: (**a**) DAFCHO; and (**b**) FDACHO.

The synthesis of two oligophenyleneimines designated as DAFCHO and FDACHO was performed using 2,5-bis(octyloxy)terephtaldehyde and 1,4-phenylenediamine or 2,7-diamino fluorene by means from mechanosynthesis (Figure 1). In both molecules, the presence of imines with endings of aldehyde-like groups was observed. The reaction products yielded 89% and 90%, respectively, after 90 min of milling; the reaction was carried out without solvents, catalysts or inert gas atmosphere.

**Figure 2:**
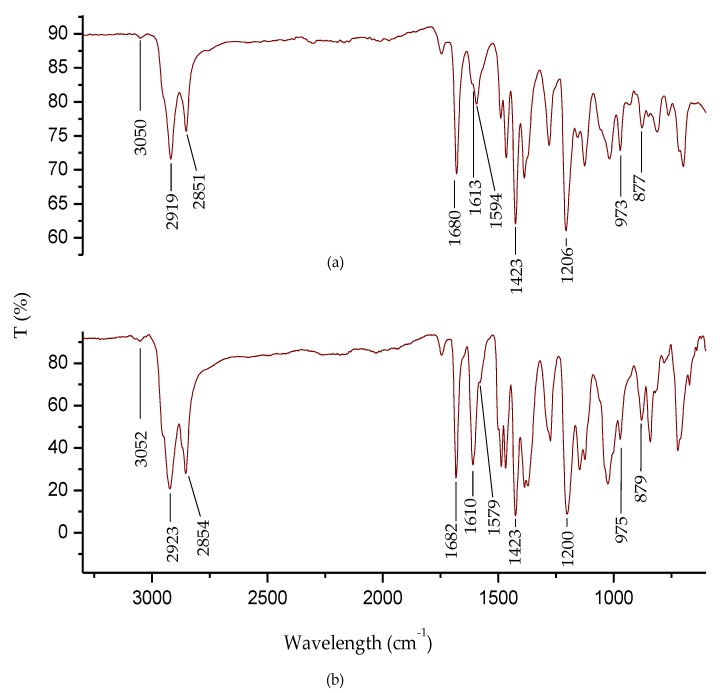
FT-IR spectra of the oligophenyleneimines: (**a**) DAFCHO; and (**b**) FDACHO.

FT-IR spectroscopy was used to characterize the DAFCHO and FDACHO oligomers (Figure 2). The results show the emergence of vibrational bands at 3050 cm^−1^ and 3052 cm^−1^, corresponding to the aromatic C-H bonds. The vibrational bands at 2919 cm^−1^, 2851 cm^−1^, 2923 cm^−1^ and 2854 cm^−1^, corresponding to the (C-H) of aldehyde (CHO), methylene (CH_2_) and methyl (CH_3_) groups, originate from the aliphatic chains. The vibrational bands at 1594 and 1579 cm^−1^ are linked to the vibration of the groups (C=C), while the vibrational bands at 1680 and 1682 cm^−1^ emerge due to the (C=O) bond, generated by the terminal aldehyde groups (CHO). Finally, the formation of the imine bonds (C=N) were observed at 1613 and 1610 cm^−1^.

**Figure 3:**
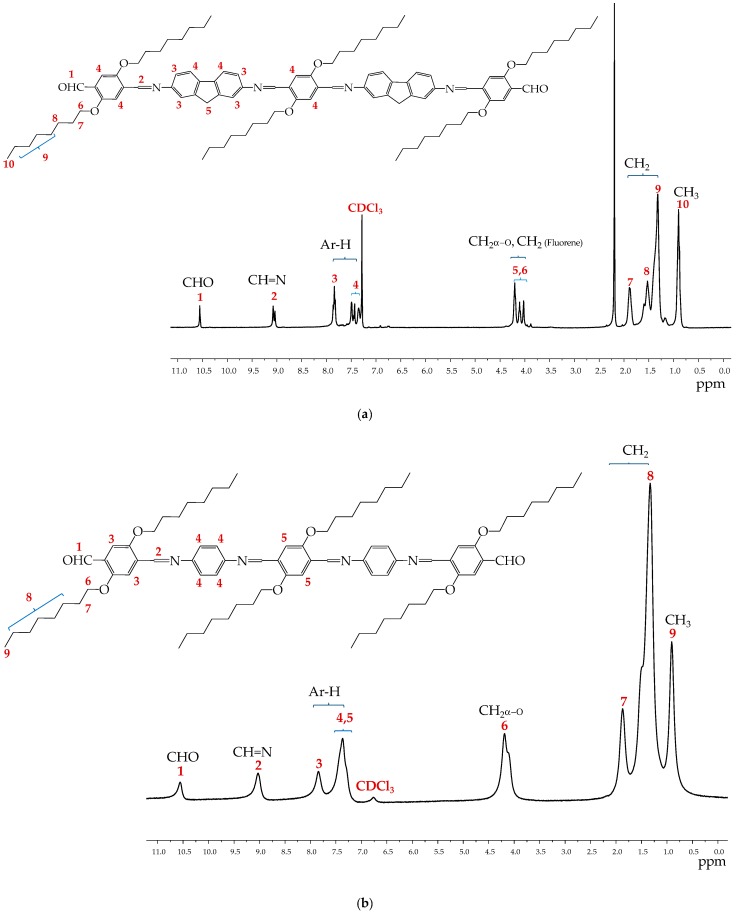
NMR-^1^H spectra in CDCl_3_ of the oligophenyleneimines: (**a**) DAFCHO; and (**b**) FDACHO.

The characterization of the oligophenyleneimines DAFCHO and FDACHO by ^1^H-NMR was performed, as shown in Figure 3. The simple signals (1) at 10.51 and 10.54 ppm correspond to protons of (CHO) aldehyde groups and integrate two protons. The broad signals (2) observed at 9.03 ppm were assigned to protons of (CH=N) imino groups and they integrate four protons. The multiple signals (3 and 4) and (3–5) that were observed in the region of 7.80–7.25 ppm correspond to (CH) protons of the aromatic groups, and integrate 18 protons for DAFCHO and 14 protons for FDACHO. The multiple signals (5 and 6) observed at 4.25–4.00 ppm correspond to (CH_2_) protons of the fluorene groups and protons (CH_2_α-O) of the methylene alpha groups of the alkoxy chain, and they integrate 16 total protons for DAFCHO. For FDACHO at 4.06 ppm, a triple signal (6) was observed, which integrates 12 protons, corresponding to the methylene alpha groups of the alkoxy chain (CH_2_α-O). The broad signals, 7 and 8 for FDACHO and 7–9 for DAFCHO were observed in the region of 1.83–1.28 ppm, integrate 72 protons, and correspond to the remaining protons of (CH_2_) methylene groups of the aliphatic chains. Finally, the simple signals that appear at 0.87 ppm integrate 18 protons, and they correspond to the terminal (CH_3_) methyl groups of the aliphatic chains, 9 for FDACHO and 10 for DAFCHO.

### 2.2. Optical Studies

#### 2.2.1. Absorption Spectra in Different Solvents

**Figure 4:**
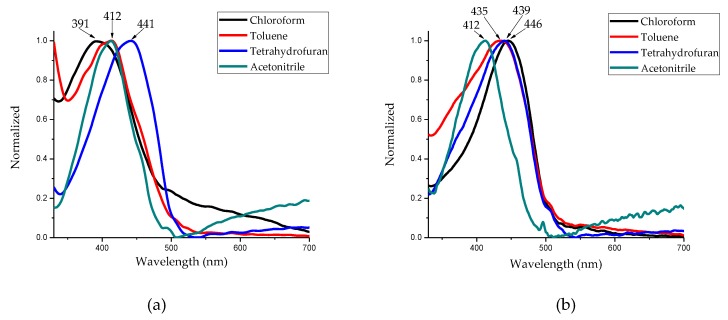
Absorption spectra: (**a**) DAFCHO (0.026 mol/mL); and (**b**) FDACHO (0.030 mol/mL) in different solvents.

The solvent effect on the optical properties for the DAFCHO and FDACHO compounds was analyzed in different solvent mediums such as chloroform, tetrahydrofuran, toluene and acetonitrile (Table 1), observing the displacements of the maximum absorption peaks (π–π* transitions). These transitions were observed for DAFCHO, (Figure 4a). For example, in tetrahydrofuran, the greatest band shift was observed (at 441 nm). In toluene and acetonitrile, the band peak shifted up to 412 nm, while, in chloroform, a shift, considered as hypsochromic, was observed at 391 nm. For FDACHO (Figure 4b), the absorption spectrum was obtained in chloroform, tetrahydrofuran, toluene and acetonitrile, peaks at 446, 439, 435 and 412 nm, respectively, were observed, showing the influence of solvents on the band peak shifts from the electronic π–π* transition. In the FDACHO and DAFCHO studies, the greatest displacement was observed on FDACHO due to the fact that the rings originated from phenylenediamine give a greater planarity to the conjugated system, favoring the intermolecular interactions that causes an increasement in absorption with decreasing optical gap. In contrast, for DAFCHO, fluorene rings having central sp^3^ carbons cause a significant decrease in the planarity of the conjugated system.

#### 2.2.2. Photochromic Properties

**Figure 5:**
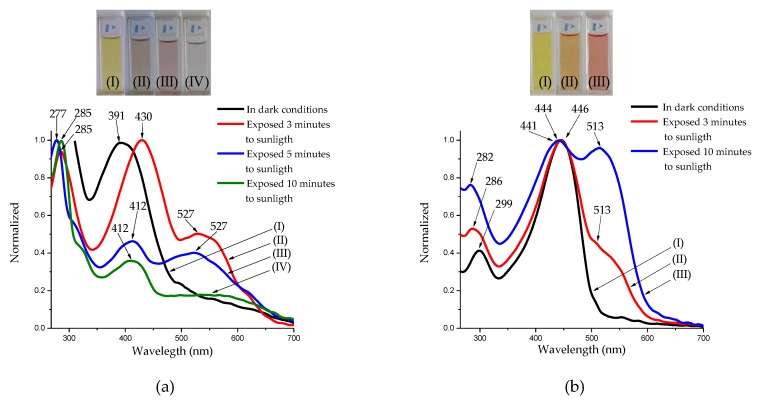
Absorption spectrum: (**a**) DAFCHO and (**b**) FDACHO in chloroform under dark conditions without irradiation (black line) and photochromic effect with irradiation of sunlight (red line, blue line and green line) at room temperature.

Interestingly, for the oligophenyleneimines DAFCHO and FDACHO, a photochromic behavior was observed in chloroform when exposed to sunlight irradiation for 3, 5 and 10 min. The change of color was monitored through their absorption spectra for both compounds. As shown in (Figure 5a), in dark conditions, DAFCHO exhibits yellow color in solution (I (a)), showing an absorption peak at 391 nm. However, after exposing the solution to sunlight for 3 min, a change to light brown (II (a)) with an absorption at 430 nm was observed. If the sunlight irradiation continued for 5 min, a new absorption peak at 527 nm presents, undergoing further color change to light pink (III (a)). This shows that there is a bathochromic shift at 412 nm with respect to the initial measurement at 391 nm but the new absorption signal at 527 nm is conserved. Finally, if the solution is irradiated with sunlight for 10 min, a colorless solution is obtained (IV (a)) with a hypsochromic shift at 407 nm. Nevertheless, the signal at 527 nm decreases, emerging a shoulder-like feature. Interestingly, when the DAFCHO solution is stored under dark conditions, the yellow color is recovered in the solution, showing that the compound possesses photochromic properties. Similarly, for FDACHO oligophenyleneimine, the studies for photochromic properties were performed (Figure 5b). The results show that the compound in chloroform exhibits a yellow color in dark conditions (I (b)) emerging an absorption peak at 446 nm. However, if the solution is irradiated with sunlight for 3 min, a change of color to orange was observed (II (b)), obtaining the signal at 444 nm, along with a new shoulder at 513 nm. When irradiating for 10 min, the color of solution becomes pink (III (b)), obtaining an absorption peak at 441 nm with a shoulder at 513 nm. As seen for DAFCHO, when FDACHO is stored in dark conditions and room temperature, the original yellow color is recovered. In both cases, the new band that emerges at 527 and 513 nm, respectively, when the compounds are irradiated with sunlight is evidence of the *cis-trans photoisomerization* in accordance with previous reports [18].

The results obtained show that both pentamers DAFCHO and FDACHO with aldehyde terminations have photochromic properties. In those with amine terminations named OIC1MS and OIC2MS [15] that we previously reported, only the pentamer produced by mechanosynthesis, with fluorene units in its structure, presents a photochromic effect. However, more studies are required to verify if this property is produced by the synthesis method, or by the structure of the oligomer under study. It is known that fluorene-containing materials are widely studied in optoelectronics because of the planarity and high conjugation promoted in the molecule [30,31].

### 2.3. Electrochemical Analysis

**Figure 6:**
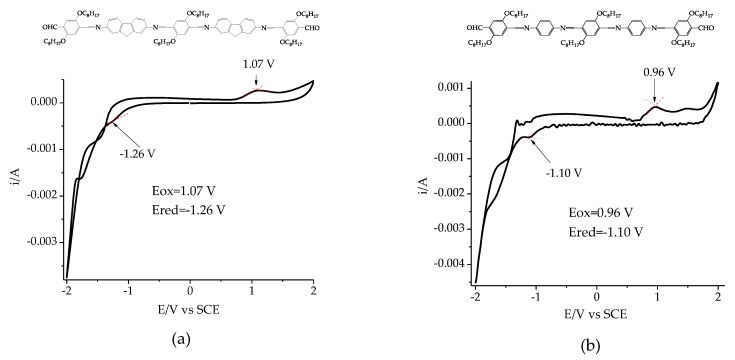
Voltammograms: (**a**) DAFCHO and (**b**) FDACHO, in the BU_4_NPF_6_/Acetonitrile system; 0.1 M, *v* = 50 mV/s.

The electrochemical properties of DAFCHO and FDACHO were analyzed by means of cyclic voltammetry, where the working electrode was coated using films of each compound. The voltammogram obtained for DAFCHO (see Figure 6a) shows that the peaks corresponding to the cathodic exploration were detected at −1.26 V and the anodic exploration at 1.07 V. In the same way, for FDACHO, the electrochemical behavior was observed (see Figure 6b) showing the reduction peak Ered at −1.10 V and the oxidation peak Eox at 0.96 V. In both cases, when the voltage changed, a color change in the film was observed, from orange to dark brown. However, this process was not reversible. Therefore, more studies should be performed to verify if these compounds present electrochromic effect. Finally, the electrochemical parameters were used to calculate their band gap energies (Eg_(EC)_) by using the reported procedure [32]. The data are summarized in Table 2. The data reveal that the presence of oxidative/reductant peaks in the voltammograms for the conjugated molecules are linked to the formation of polarons that corresponds to the existence of the different functional groups. The oxidation potential observed for DAFCHO is higher (+1.07) than that detected for FDACHO (+0.96). However, the reduction potential obtained for DAFCHO is relatively lower (−1.26) than that exhibited by FDACHO (−1.10). According to previous studies [32], the introduction of the imine groups into the conjugated structure promotes more active sites for the reduction process, because of unbonded pairs of nitrogen electrons, but the aldehyde groups promotes the oxidation process.

### 2.4. Theoretical Study

**Figure 7:**
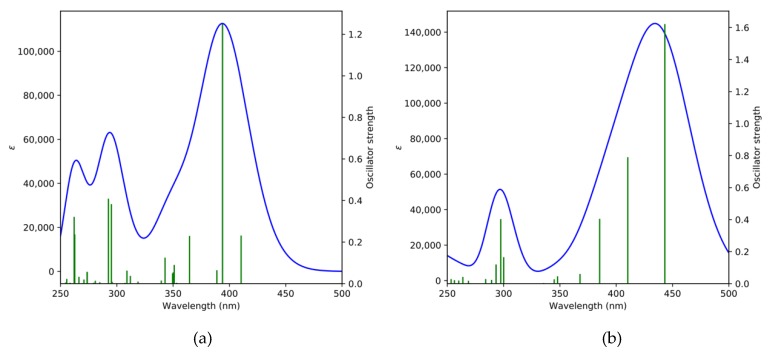
Simulated UV-Vis spectra of DAFCHO (**a**) and FDACHO (**b**) calculated at the TD-M062x/cc-pVTZ level of theory with PCM parameters for chloroform using FWHM (Full Width at Half Maximum) of 3000. Individual excitations are displayed as vertical lines with heights representing their oscillator strengths.

The calculated theoretical spectra reproduce remarkably well the experimental spectra in both shape and position of the maxima, as shown in Figure 7, with the main peak of DAFCHO located around 394 nm and the main peak of FDACHO located around 443 nm. In order to characterize the orbital contributions that correspond to each peak, the main electronic excitations (oscillator strength greater than 0.2) are reported in Table 3. For each excitation the major orbital contributions are listed. 

For DAFCHO the most intense excitation is observed around 394 nm and is related to the HOMO→LUMO+2 transition, whose contribution to the first excited state is around 51%, while the HOMO−1→LUMO transition contribute another 13% (see Table 3). For FDACHO the most intense excitation was observed around 443 nm being the HOMO→LUMO transition its major contribution with 62% while the HOMO−1→LUMO+1 transition contributing another 12%.

**Figure 8:**
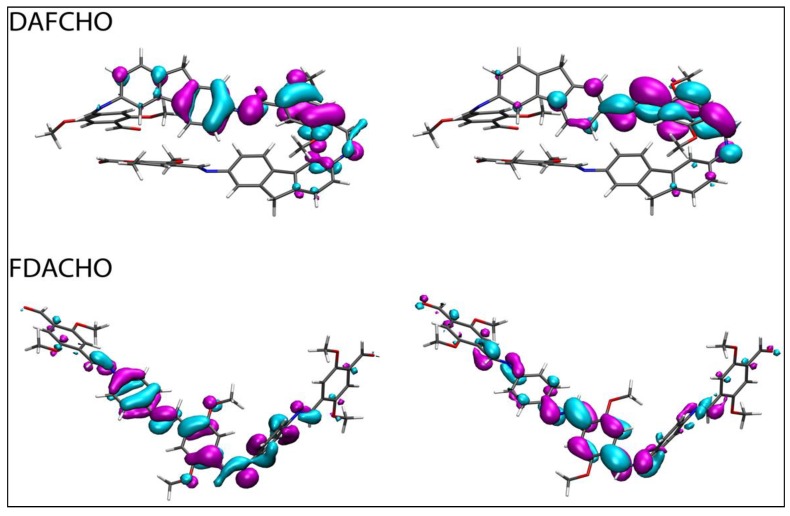
Isosurfaces of the frontier NTOs representing the hole (left) and particle (right) spatial location for the second electronic transition of DAFCHO and the first electronic transition of FDACHO.

Furthermore, the main electronic transitions of both molecules were analyzed through natural transition orbitals (NTOs) [33] calculated at the M062X/cc-pVTZ level of theory. Figure 8 displays the resultant NTOs isosurfaces, showing that there is a common nodal surface separating the two phases of the HOTO and LUTO orbitals, as is characteristic of π→π* transitions [34]. For DAFCHO the hole is localized over the inner phenyl ring and part of the fluorene groups while the particle is transferred to the inner phenyl ring. For FDACHO the hole is localized over the three inner phenyl rings and the oxygen of the central phenyl group while the particle is transferred over the two chains bridging the three inner phenyl rings.

## 3. Experimental

### 3.1. General

All chemicals were purchased at Sigma-Aldrich (St. Louis, MO, USA) and used as received and the solvents at J.T. Baker (Phillipsburg, NJ, USA). The NMR spectra were recorded in CDCl_3_ with tetramethylsilane (TMS) as an internal reference on an Agilent Varian instrument (400 MHz for ^1^H and 75 MHz for ^13^C, (Santa Clara, CA, USA). The FT-IR (ATR) spectra were measured on a Perkin-Elmer Frontier spectrophotometer (Waltham, MA, USA). MALDI-TOF mass spectra were taken on a Bruker Daltonic flexAnalysis (Billerica, MA, USA). The UV-Vis spectra were measured on a Perkin-Elmer Lambda XLS spectrometer (Waltham, MA, USA). For the emission spectra, a Perkin Elmer LS55 spectrometer (Waltham, MA, USA).) was used and the excitation wavelength was chosen at 10 nm under the absorption peak. The films were deposited on glass slides by self-assembly.

### 3.2. Mechanochemistry

Mechanochemistry of DAFCHO and FDACHO were carried out in a mixer mill Spex 8000D (Metuchen, NJ, USA) in D2 tool steel vials with hardened steel balls. 2,5-bis(octyloxy)terephtal aldehyde (0.100 g., 0.2560 mmol) and 1,4-phenylendiamine (0.012 g., 0.1164 mmol) or 2,5-diamino fluorene (0.020 g., 0.1024 mmol) were introduced in the steel vial and milled for 90 min. The vial was left to reach room temperature and then opened. The compound was dissolved in CHCl_3_, washed three times with water and the organic phase was dried with Na_2_SO_4_. The crude product was purified by precipitation using hexane and vacuum filtered to obtain an orange powder in 89% and 90% yield, respectively.

4,4′-((((((2,5-bis(octyloxy)-1,4-phenylene)bis(methanylylidene))bis(azanylylidene))bis(9H-fluorene-7,2-diyl))bis(azanylylidene))bis(methanylylidene))bis(2,5-bis(octyloxy)benzaldehyde).

(DAFCHO): Yield 89% (0.067 g.); orange powder; Td 247.6 °C. FT-IR/ATR ν (cm^−1^): 3050, 2919, 2851, 1680, 1613, 1594, 1423, 1206, 973, 877; ^1^H-NMR (CDCl_3_) ppm: 10.51 (s, 2H, CH=O), 9.03 (b, 4H, HC=N), 7.8-7.25 (m, 18H, Ar), 4.25–4.00 [m, 16H (4H, CH_2_-Fluorene and 12H, CH_2_α-O)], 1.83–1.28 (b, 12H, CH_2_), 1.60–1.49 (b, 12H, CH_2_), 1.45–1.25 (b, 48H, CH_2_), 0.87 (s, 18H, CH_3_); ^13^C-NMR (CDCl_3_) ppm: 198.95 (CH=O), 160.22 (C=N), 144.67 (Ar), 142.51 (Ar), 144.08 (Ar), 121.71 (Ar), 106.23 (Ar), 64.51 (CH_2_α-O), 31.82 (CH_2_), 30.93 (CH_2_) 29.32 (CH_2_), 26.12 (CH_2_), 22.67 (CH_2_), 20.14 (CH_2_), 14.10 (CH_3_); MS (*m*/*z*): 1496 [M^+^]. (Supplementary Materials).

4,4′-((((((2,5-bis(octyloxy)-1,4-phenylene)bis(methanylylidene))bis(azanylylidene))bis(4,1-phenylene))bis(azanylylidene))bis(methanylylidene))bis(2,5-bis(octyloxy)benzaldehyde).

(FDACHO): yield 90% (0.065 g.); orange powder; Td 246.2 °C. FT-IR/ATR ν (cm^−1^): 3052, 2923, 2854, 1682, 1610, 1579, 1423, 1200, 975, 879; ^1^H-NMR (CDCl_3_) ppm: 10.6 (s, 2H, CH=O), 9.03 (b, 4H, HC=N), 8.0–7.2 (m, 14H, Ar), 4.2 (b, 12H, CH_2_α-O), 2.1–1.75 (b, 12H, -CH_2_), 1.6–1.1 (b, 60H, -CH_2_), 0.85 (s, 18H, CH_3_); ^13^C-NMR (CDCl_3_) ppm: 189.47 (C=O), 155.78 (C=N), 129.31 (Ar), 122.18 (Ar), 115.5 (Ar), 115.28 (Ar), 111.65 (Ar), 114.53 (Ar), 110.76 (Ar), 69.27 (CH_2_α-O), 31.78 (CH_2_), 30.93 (CH_2_), 29.33 (CH_2_), 29.24 (CH_2_), 26.08 (CH_2_), 26.02 (CH_2_), 14.10 (CH_3_); MS (*m*/*z*): 1316 [M^+^]. (Supplementary Materials).

### 3.3. Electrochemical

A typical three-electrode cell was used to carry out the cyclic voltammetry. The working glassy carbon electrode was coated with conjugated oligophenylene imine (concentration of 0.5 mg/mL in chloroform). The reference electrode was SCE, and Pt was used as counter electrode. First, the electrodes were evaluated in an electrolytic medium of 0.1M BU_4_NPF_6_ dissolved in acetonitrile. The solution was deoxygenated using high purity nitrogen for 30 min before performing electrochemical experiments. The cathodic potential sweeps were recorded in the intervals of −2 V to 2 V vs. SCE, at a speed of 50 mV/s at room temperature.

### 3.4. Computational

Multiple molecular dynamic simulations of DAFCHO and FDACHO were performed with DFTB+ software (version, 17.1, Bremen, Germany) [35], cooling the system using a linear schedule from 1500 K to 1 K in 25,000 time-steps of 2.0 fs. The three most stable conformers of each molecule were then locally optimized at the DFT LSDA/6-31G* level of theory, correcting for solvent effects with the polarizable continuum model (PCM) with parameters for chloroform. For each conformer, the electronic excitations were calculated at the TD-DFT theory M062X/cc-pVTZ/PCM level of theory. The UV/vis spectrum was generated with a convolution of Gaussian functions with the first 30 electronic excitations with the aid of the GaussSum package [36]. All DFT and TD-DFT calculations were performed with the Gaussian 09 [37] software (version 09 rev. C, Wallingford, CT, USA). The UV/visible spectra of DAFCHO and FDACHO were calculated substituting the aliphatic lateral chains by a methyl group, leaving the backbone with a methoxy group. This simplification only affects slightly the electronic transitions as they occur in the aromatic backbone [15].

## 4. Conclusions

In the present work, it was demonstrated that mechanosynthesis is an efficient, selective, and environmentally friendly method for the synthesis of oligophenyleneimine type pentamers. The products DAFCHO and FDACHO exhibit interesting photochromic properties. This is probably caused by reversible structural rearrangements generated by the photoisomerization when the compounds are excited by irradiation with sunlight in accordance with other reports but in this work the photochromic effect lasts longer than those reported by other authors. The *cis-trans photoisomerization* leads to a charge transfer phenomenon that was observed as new transitions at higher wavelength, but disappeared when the compounds are keep in dark conditions. After analyzing optical and electrochemical bandgap, the oligophenyleneimines behave as semiconductors, making them candidates for the manufacture of optoelectronic devices. DFT calculations showed that the electronic excitations in these molecules cannot be described by single orbital transitions; nevertheless, absorption in FDACHO can be roughly seen as a HOMO–LUMO transition while absorption in DAFCHO can be related to a HOMO–L+1 transition, explaining the difference between their maximum absorption wavelengths. The thermal stability of compounds is above 240 °C.

## Figures and Tables

**Table 1:** Optical properties of DAFCHO and FDACHO in different solvents.

	DAFCHO			FDACHO		
Solvents	λ max (nm)	ε × 10^3^ (M^−1^ cm^−1^)	Eg (eV)	λ max (nm)	ε × 10^3^ (M^−1^ cm^−1^)	Eg (eV)
Chloroform	391	9.06	2.50	446	25.60	2.41
Tetrahydrofuran	441	16.69	2.46	439	13.62	2.43
Toluene	412	17.01	2.47	435	20.11	2.43
Acetonitrile	412	4.53	2.56	412	2.37	2.55

Band gaps were calculated from the onset of absorption spectra of the oligomer solutions [29].

**Table 2:** Electrochemical data from DAFCHO and FDACHO.

OLIGOIMINE	E_ox_ V	−E_red_ V	HOMO eV	LUMO eV	Eg_(EC)_ eV
**DAFCHO**	1.07	1.26	3.54	5.87	2.35
**FDACHO**	0.96	1.10	3.69	5.75	2.06

HOMO − LUMO were calculated according to the empirical formula [29].

**Table 3:** Main electronic excitations of molecules DAFCHO and FDACHO, listing only their major molecular orbital contributions (greater than 10%).

Transition	Energy (eV)	Wavelength (nm)	Osc. Strength	Major Contribs
**DAFCHO**				
**2**	3.15	393.89	1.2490	H−1→LUMO (13%) HOMO→L + 2 (51%)
**17**	4.24	292.55	0.4089	H−2→LUMO (31%)
**16**	4.20	295.18	0.3828	H−2→LUMO (32%)
**27**	4.73	262.11	0.3215	H−1→L + 4 (31%)
**26**	4.72	262.66	0.2376	H−11→L + 1 (11%) H−10→L + 1 (13%) H−1→L + 4 (20%)
**1**	3.02	410.38	0.2316	H−1→LUMO (56%) HOMO→L + 2 (14%)
**4**	3.40	364.57	0.2299	H−4→L + 1 (14%) H−3→LUMO (21%) H−3→L + 1 (36%)
**FDACHO**				
**1**	2.80	443.16	1.6216	H−1→L + 1 (12%) HOMO→LUMO (62%)
**2**	3.02	410.28	0.7902	H−1→LUMO (53%) HOMO→L + 1 (19%)
**3**	3.22	385.28	0.4051	H−1→L + 1 (17%) H−1→L + 2 (16%) HOMO→L + 1 (22%) HOMO→L + 2 (13%)
**11**	4.16	297.49	0.4037	H−14→LUMO (11%) H−9→LUMO (19%)

## Supplementary Materials

The Supplementary Materials are available online.
