# Supplementary material for: Mechanosynthesis of Photochromic Oligophenyleneimines: Optical, Electrochemical and Theoretical Studies
Source: Molecules. 2019 Feb 28;24(5):849. doi: 10.3390/molecules24050849 (PMC6429622; doi:10.3390/molecules24050849)

Supplementary File

Fragment Patron DAFCHO

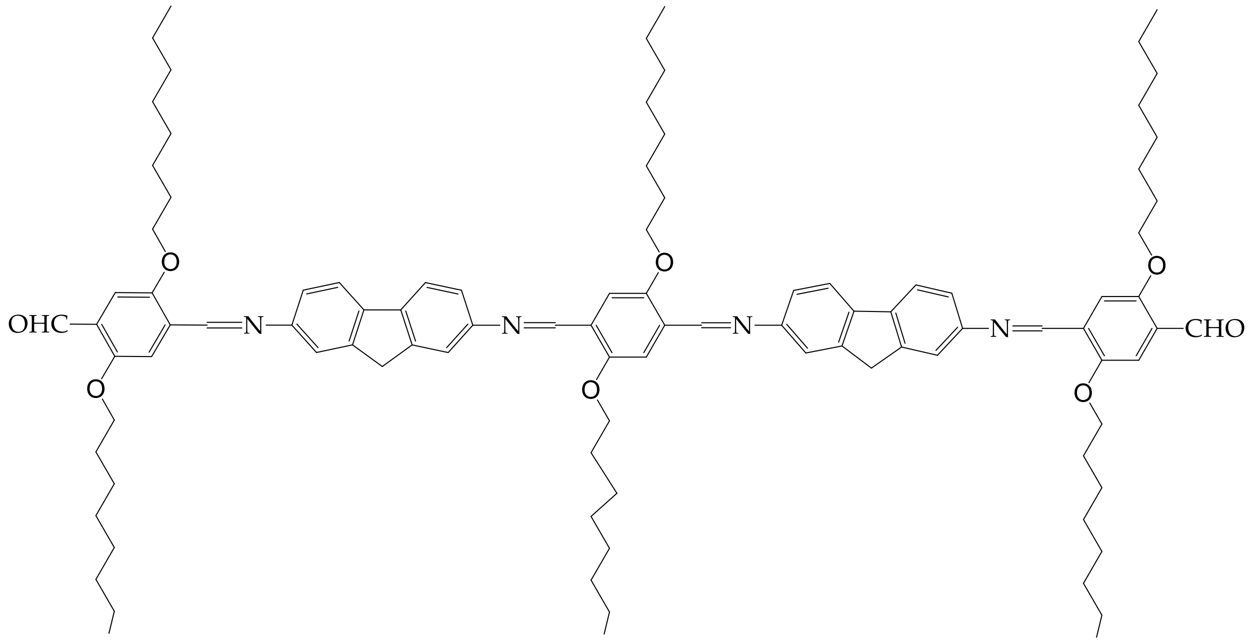

m/z: 1491.99 (100.0%), 1490.99 (92.8%), 1493.00 (53.4%), 1494.00 (20.5%), 1495.00 (5.8%), 1492.99 (3.0%), 1492.00 (1.4%), 1496.01 (1.0%)

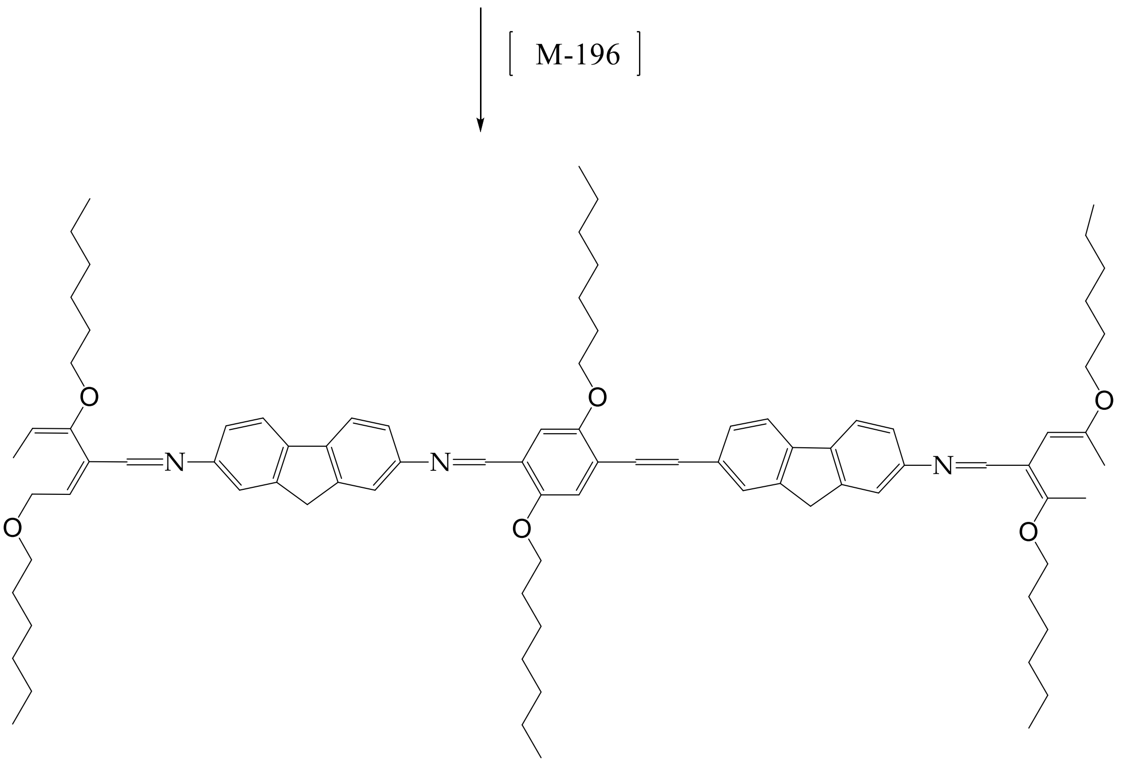

m/z: 1301.91 (100.0%), 1302.91 (95.4%), 1303.92 (45.3%), 1304.92 (15.3%), 1305.92 (3.8%), 1303.91 (2.3%), 1302.92 (1.4%)

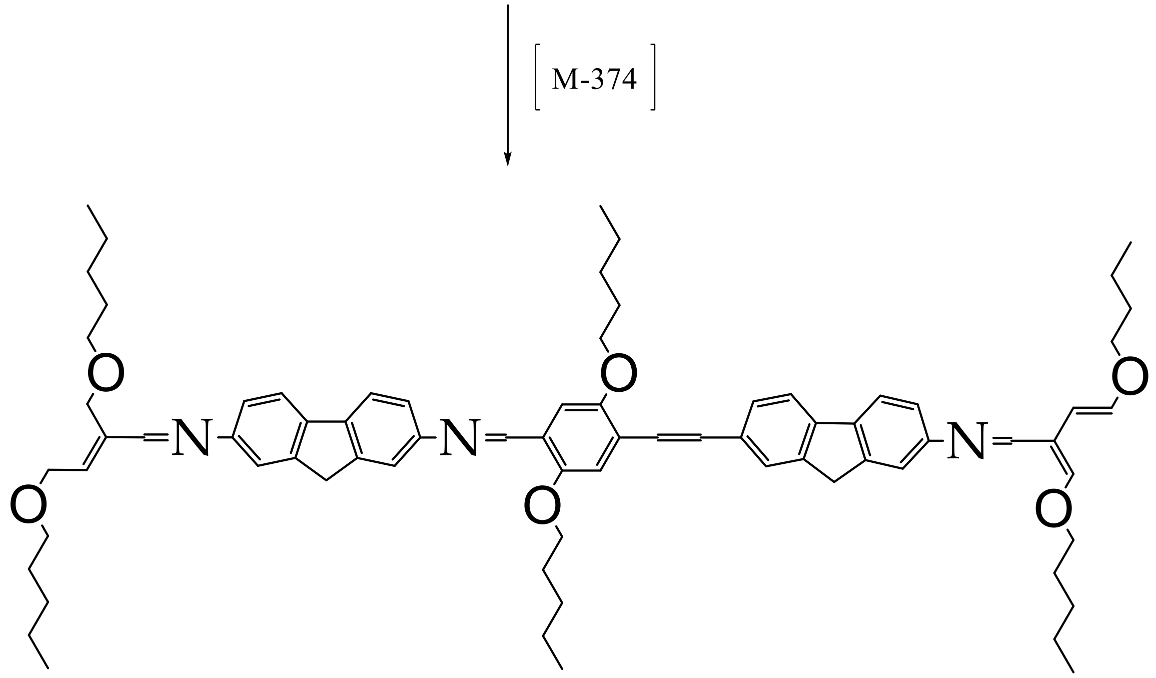

m/z: 1121.72 (100.0%), 1122.73 (81.4%), 1123.73 (33.9%), 1124.73 (9.6%), 1125.74 (1.7%), 1122.72 (1.1%)

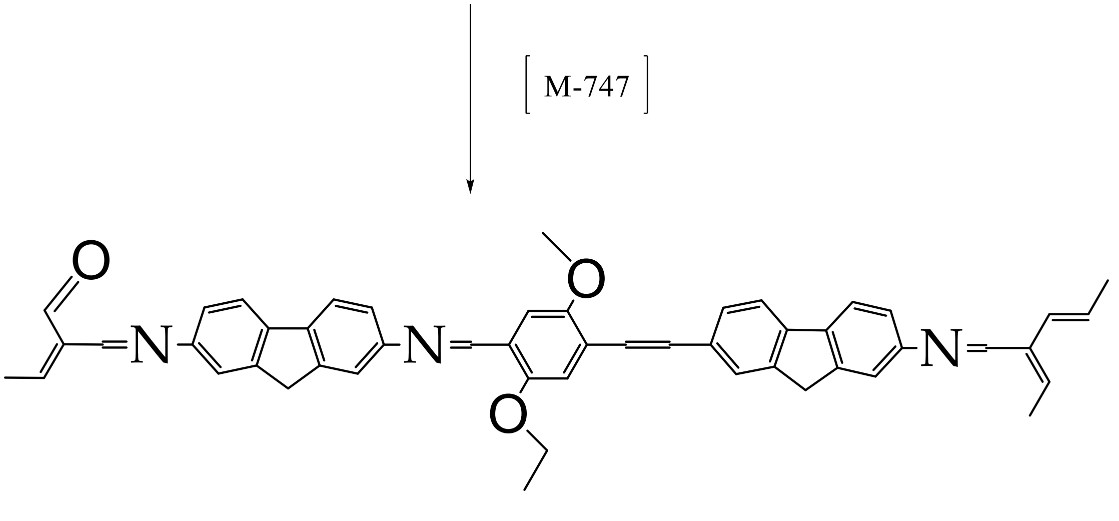

m/z: 749.36 (100.0%), 750.37 (55.8%), 751.37 (15.9%), 752.37 (3.2%), 750.36 (1.1%)

Fragment Patron DAFCHO

Fragment Patron FDACHO

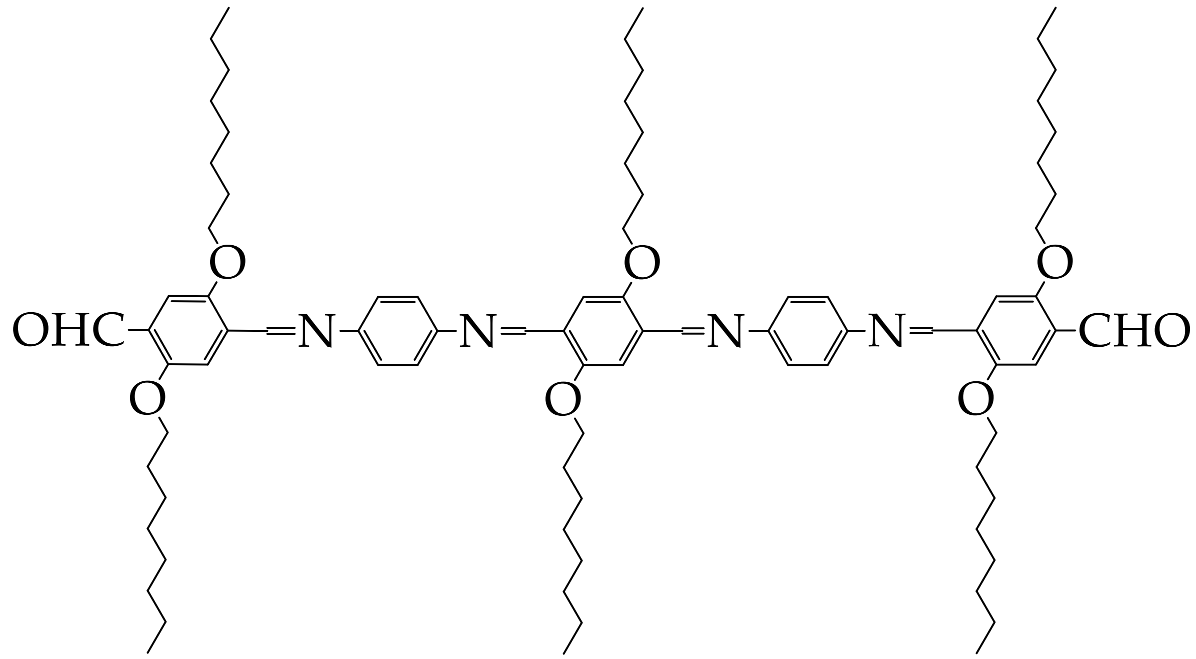

m/z: 1314.93 (100.0%), 1315.93 (92.6%), 1316.93 (44.1%), 1317.94 (12.8%), 1318.94 (3.5%), 1317.93 (2.1%), 1315.92 (1.5%), 1316.94 (1.3%)

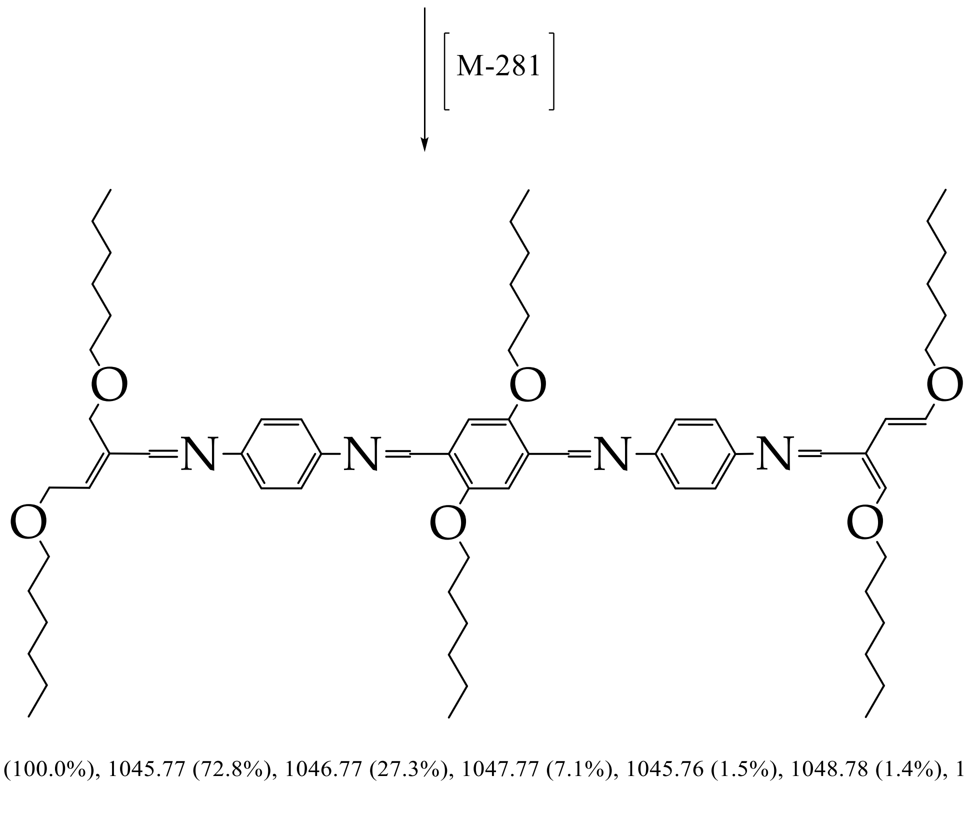

m/z: 1044.76 (100.0%), 1045.77 (72.8%), 1046.77 (27.3%), 1047.77 (7.1%), 1045.76 (1.5%), 1048.78 (1.4%), 1046.76 (1.1%)

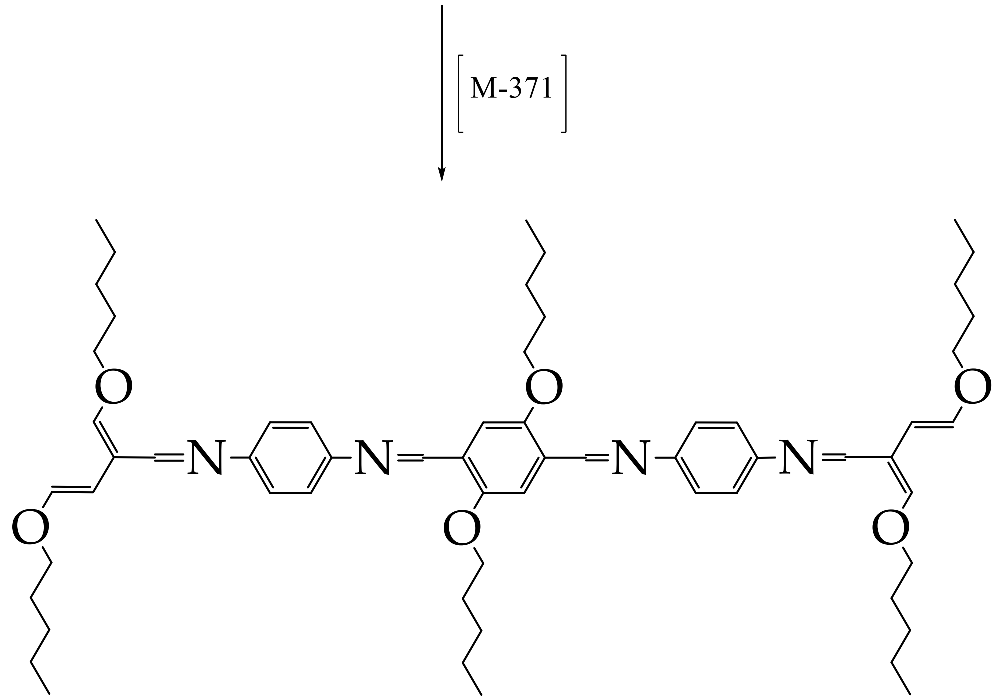

m/z: 958.65 (100.0%), 959.66 (66.1%), 960.66 (23.7%), 961.66 (5.5%), 959.65 (1.5%)

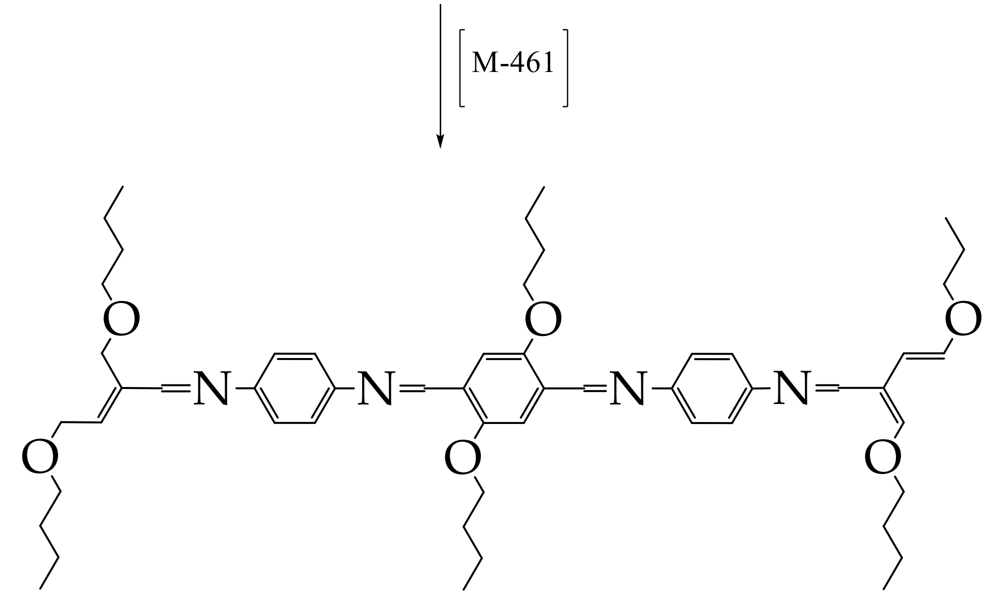

m/z: 862.56 (100.0%), 863.56 (58.8%), 864.57 (18.0%), 865.57 (3.9%), 863.57 (1.1%)

Fragment Patron FDACHO

MALDI-TOF mass spectra of DAFCHO

Registro DAFCHO 171127-mgm-03
Matriz DHB 2/5

Laboratorios de Servicios Analiticos
Instituto de Quimica UNAM

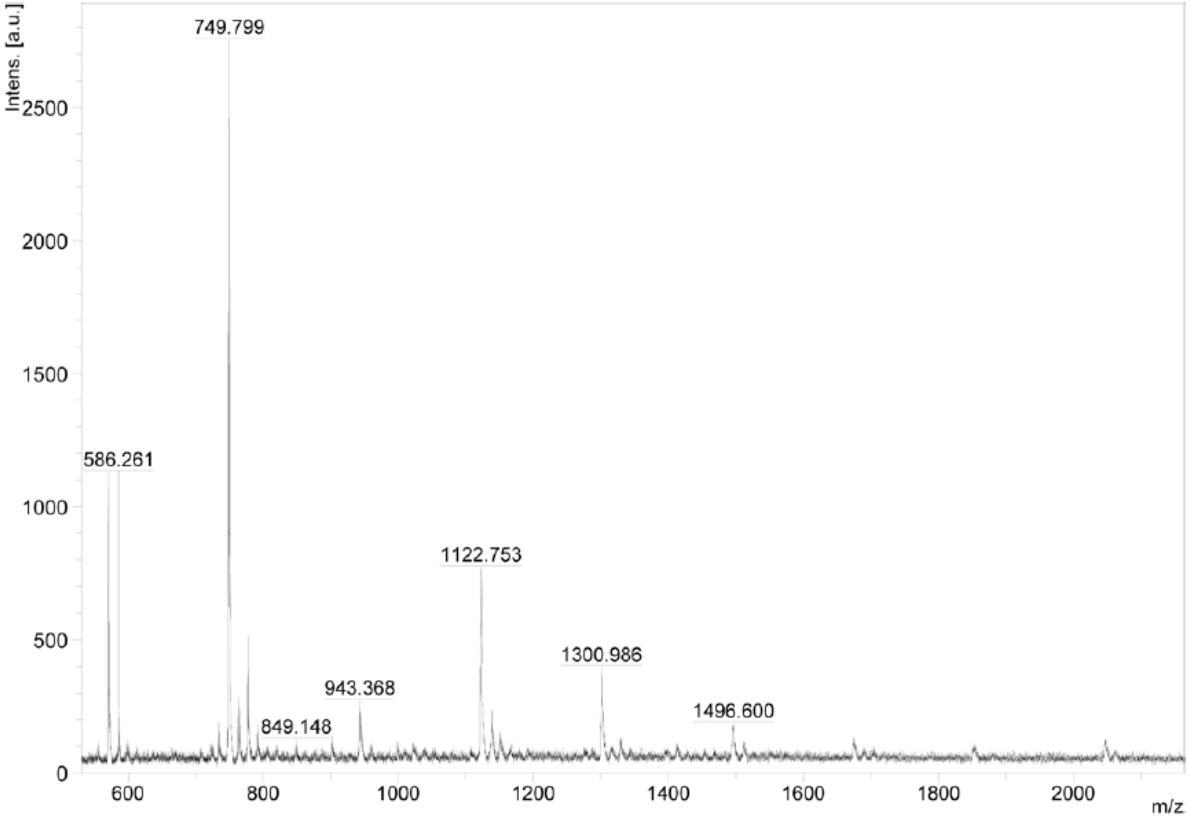

Acquisition Parameter

Date of acquisition	2017-12-11T11:37:14.531-06:00
Acquisition method name	D:\Methods\flexControlMethods\LNM_UNAM\LP_PepMix.par
Aquisition operation mode	Linear
Voltage polarity	POS
Number of shots	50
Name of spectrum used for calibration	
Calibration reference list used	

Instrument Info

User	UNAM
Instrument	FLEX-PC
Instrument type	microflex

D:\data\LSA\DAFCHO 171127-mgm-03\0_F8\1

Bruker Daltonics flexAnalysis printed: 12/11/2017 11:45:19 AM

MALDI-TOF mass spectra of FDACHO

Registro FDACHO 171127-mgm-12
Matriz DHB 2/5

Laboratorios de Servicios Analiticos
Instituto de Quimica UNAM

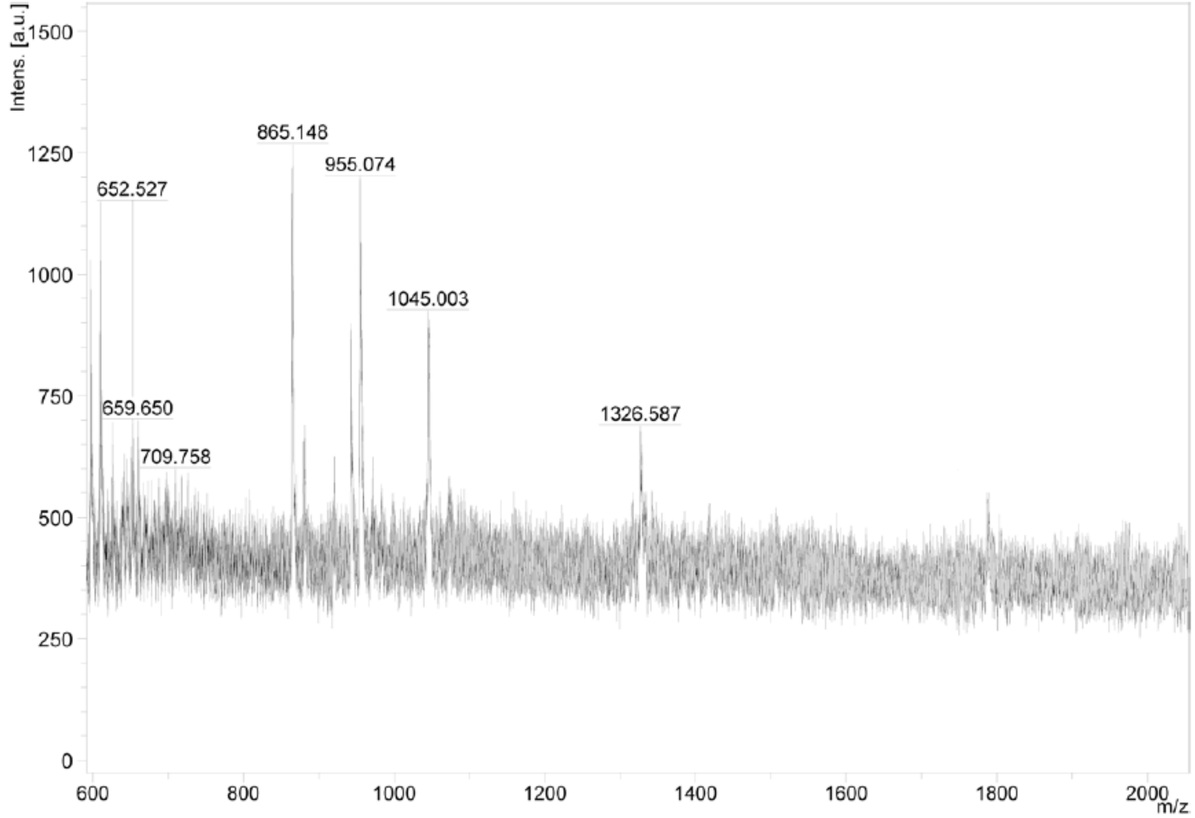

Acquisition Parameter

Date of acquisition	2017-12-11T13:55:37.109-06:00
Acquisition method name	D:\Methods\flexControlMethods\LNM_UNAM\LP_PepMix.par
Aquisition operation mode	Linear
Voltage polarity	POS
Number of shots	400
Name of spectrum used for calibration	
Calibration reference list used	

Instrument Info

User	UNAM
Instrument	FLEX-PC
Instrument type	microflex

D:\data\LSA\FDACHO 171127-mgm-12\0_D10\1

Bruker Daltonics flexAnalysis printed: 12/11/2017 3:21:56 PM

DAFCHO-IH-NMR

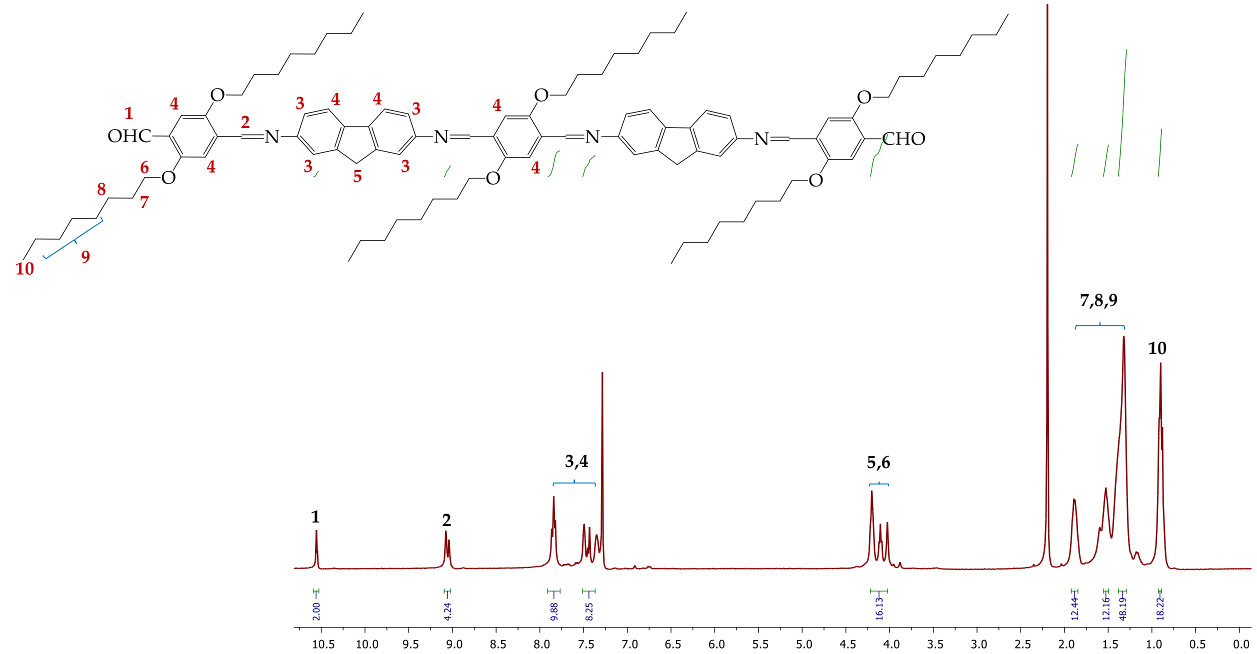

FDACHO-IH-NMR

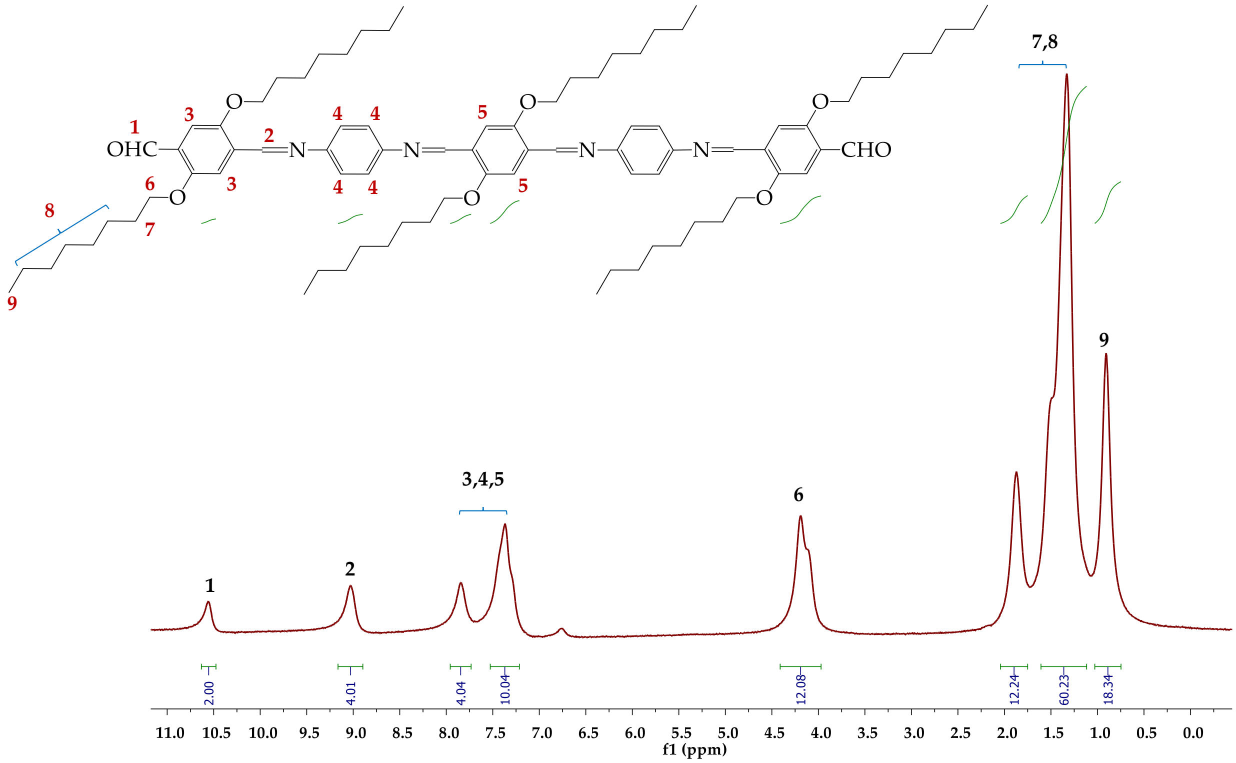

DAFCHO-TGA

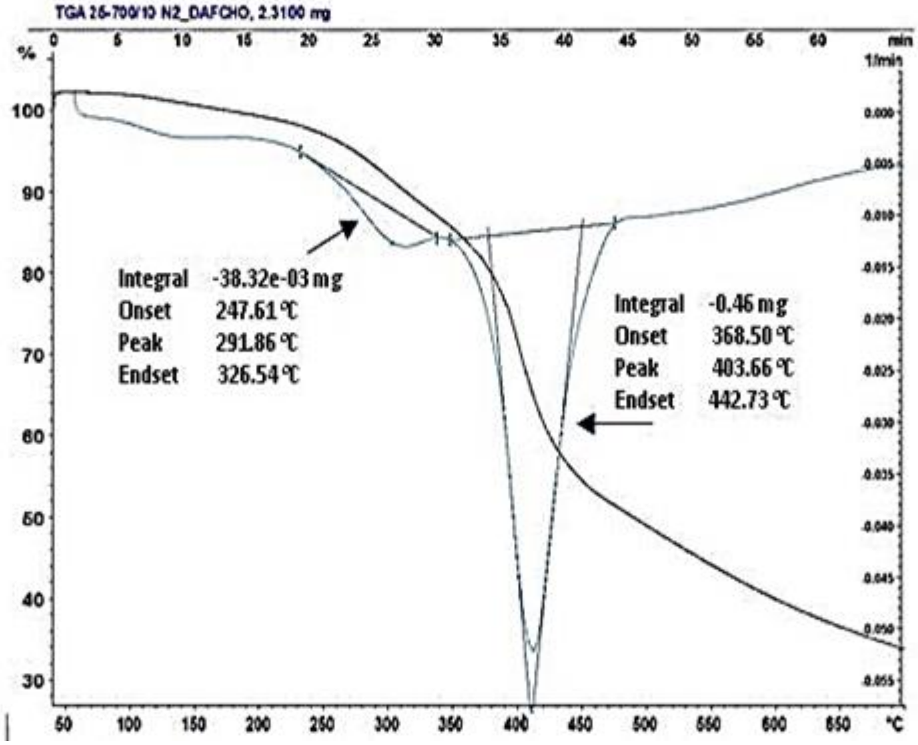

DAFCHO-TGA

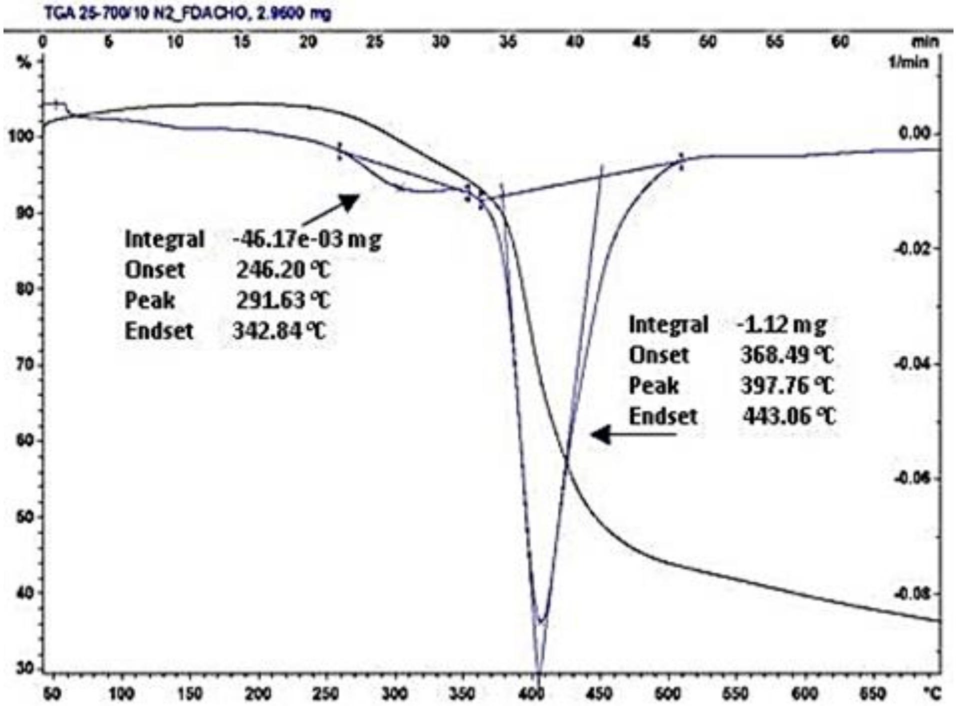

Supplement: Supplementary file 1 [file molecules-24-00849-s001.pdf]
